# Corticosteroids in severe COVID-19: a critical view of the evidence

**DOI:** 10.1186/s13054-020-03360-0

**Published:** 2020-10-29

**Authors:** Daniel De Backer, Elie Azoulay, Jean-Louis Vincent

**Affiliations:** 1 Department of Intensive Care, CHIREC Hospitals, Université Libre de Bruxelles, Brussels, Belgium; 2 Medical Intensive Care Unit, Saint Louis University Hospital, APHP, Paris, France; 3 Department of Intensive Care, Erasme University Hospital, Université Libre de Bruxelles, Route de Lennik 808, 1070 Brussels, Belgium

Since December 2019, SARS-CoV-2 has infected millions of people worldwide, causing excess deaths and a surge in demand for ICU beds. With no effective therapies against SARS-CoV-2, randomized trials of several potential therapeutic agents, including steroids, have been conducted. Although use of steroids in patients with ARDS [1] and severe viral pneumonia [2, 3] has been challenged, several arguments support the biological plausibility of steroid use in patients with severe COVID-19. First, autopsy studies in COVID-19 patients showed lymphocyte alveolitis, acute fibrinous injury and organizing pneumonia [4], which are all probably steroid-sensitive. Second, COVID-19 leads to activation of endothelial cells causing not only systemic inflammation but also microvascular thrombosis, pulmonary infarcts and venous thromboembolism [4, 5]. Admittedly, there are also arguments against steroid use. First, viral particles are often found at autopsy [4], and steroids may decrease viral clearance. Second, steroids only influence the inflammatory component of the inflammation–thrombosis–hypoxia interaction [6], suggesting that steroids may be less effective once thrombi have developed.

The RECOVERY trial compared administration of 6 mg/day dexamethasone for 10 days to usual care in 6425 hospitalized patients with SARS-CoV-2 infection. Survival was significantly higher in the dexamethasone-treated patients, especially in the subgroup of 1007 patients receiving invasive mechanical ventilation [7]. As a result of the RECOVERY findings, three further steroid trials, focusing on ICU patients, were stopped prematurely after inclusion of 384 [8], 299 [9] and 149 [10] patients, respectively. A meta-analysis of the available data concluded that administration of systemic steroids was associated with a decrease in 28-day mortality [11]. Nevertheless, although administration of steroids appears promising, several limitations must be considered when interpreting the results (Table 1).

**Table 1:** Limitations in the RECOVERY trial and the meta-analysis

Study	Limitations
RECOVERY trial [7]	No stratification between centers
	BMI, ethnicity not reported
	Location of patient at randomization unknown (ward/ICU)
	Age imbalance in the study population
	Distribution of the various factors associated with outcome not reported for the different subgroups
	For patients receiving mechanical ventilation, PEEP, FiO_2_, PaO_2_/FiO_2_ not reported
	Short-term outcome (28-day mortality) reported
Meta-analysis [11]	Selected data only were included
	Some of the trials were stopped prematurely and underpowered
	Excessive weight (57%) of a single trial (RECOVERY) with its own limitations see above
	Imbalance in age and sex in favor of dexamethasone in the CODEX trial, which accounted for 19% of weight
	Imbalance in age and BMI in favor of hydrocortisone in the CAPE COVID trial which accounted for 7% of weight
	Selected patients from the REMAP CAP trial were included, increasing the mortality difference from 2 to 7%. This trial accounted for 12% of weight
	Conflicting results for the subgroups of mechanical ventilation versus no ventilation between meta-analysis and RECOVERY trial
	Short-term outcomes only considered (21- to 28-day mortality)

## Critique of the evidence

### RECOVERY trial

Most multicenter randomized trials stratify by center to minimize potential center bias, but this was not done in the RECOVERY trial [7]. Although no information on center allocation in each group is provided, as more than 170 centers participated in the trial for a total of 6425 patients it is likely that center imbalance in group allocation may have occurred, especially in the subgroup of patients receiving mechanical ventilation in which an average of only 6 patients were included per center. As case fatality rates vary across hospitals [12, 13], this imbalance may have influenced the results, all other factors being equal. Indeed, in the UK, where the RECOVERY trial was performed, the magnitude of the risk of death varied between centers from 0 to over + 4, similar to the impact of age [14].

Moreover, several factors known to contribute to mortality in COVID-19 were not measured, including ethnicity and obesity. For patients receiving mechanical ventilation, severity of hypoxemia, ventilator settings and other types of organ support were not reported, yet are associated with outcome. For those factors that were measured, their frequencies in the two study groups were not reported for the subgroups. The possibility of an imbalance between the groups therefore cannot be excluded.

### The meta-analysis

The strength of this meta-analysis [11] is the inclusion of individual trial data, although not all data from the original trials were used. Because of different definitions of “critically ill,” only data from the patients who received mechanical ventilation, in whom steroids are known to have the greatest effect, were included from the RECOVERY trial. The other trials also included patients who did not receive mechanical ventilation, so one would have liked to also see these data from the RECOVERY trial. Surprisingly, a secondary analysis, focusing on mechanical ventilation, indicated that corticosteroid use was of greater benefit in non-ventilated [OR 0.41 (0.19–0.88)] than in ventilated [OR 0.69 (0.55–0.86)] patients (*p* = 0.0084), even though the RECOVERY trial contributed 57% of the weight of the meta-analysis, and the trial results seemed to show a greater benefit in ventilated than in non-ventilated patients.

Moreover, from the three trials stopped prematurely [8–10], the meta-analysis only included patients if they were randomized before the RECOVERY results were published, with the rationale that this may have influenced the management of these later patients. However, removing some patients decreased the weight of two of these trials, which had lower point estimates than those of the RECOVERY trial, but did not affect the weight of the third, because no further patients were included. More importantly, the mortality rate of the steroid-treated patients excluded from the REMAP-CAP trial [8] was markedly lower than that of the total trial population (41 patients were removed; mortality in the first 197 patients was 24.8% for hydrocortisone vs. 31.5% for controls compared to 29.9% and 32.7%, respectively, in the total 238 patients); restricting inclusion to the first 197 patients therefore clearly favored use of corticosteroids.

## Is 28-day mortality the best endpoint in COVID-19 patients?

Assessing outcome at 28 days may not be optimal for several reasons. In a randomized trial of corticosteroids in patients with ARDS, short-term variables improved with steroids, but there was no decrease in ventilator-free days at day 180 and even an increase in 90-day mortality in some subgroups [1]. Moreover, patients with severe COVID-19 often require prolonged ICU and hospital stays, beyond day-28, especially when receiving invasive mechanical ventilation [15]. In the REMAP-CAP study [8], the median duration of ICU stay was around 24 days and about 30% of the patients were still in the ICU at day 90. Finally, many patients develop secondary viral, bacterial or fungal infections, which may impact prognosis; these may occur after day 28 and thus not be apparent in earlier analyses.

## Conclusions and perspectives

The RECOVERY trial results suggest that corticosteroids may be beneficial in patients with COVID-19, especially when mechanical ventilation is required. However, the absence of stratification and incomplete information about some factors associated with outcome may have resulted in imbalance between the treated and control groups. While the meta-analysis points toward a beneficial effect of corticosteroids, it also has limitations, precluding definitive conclusions. Finally, beneficial effects at the relatively short day-28 mortality endpoint may not translate into longer-term benefit. Studies to assess longer term physical and functional outcomes in critically ill COVID-19 patients are warranted.

## Data Availability

Not applicable.

## Abbreviations

ARDS: Acute respiratory distress syndrome
COVID-19: Coronavirus disease 2019
ICU: Intensive care unit
SARS-CoV-2: Severe acute respiratory syndrome coronavirus 2
